# Should Cell Culture Platforms Move towards EV Therapy Requirements?

**DOI:** 10.3389/fimmu.2015.00008

**Published:** 2015-01-28

**Authors:** Ana Aiastui

**Affiliations:** ^1^Biodonostia Health Research Institute, Cell Culture Platform, Donostia-San Sebastián, Spain

**Keywords:** cell culture platform, extracellular vesicle, cell therapy, exosomes, GMP conditions, EV quality service

Cells *talk* to each other, but do members of the scientific community communicate with each other? Do molecular and cellular biologists speak with clinicians? In the last few years, numerous research centers have been located close to hospitals leading toward a more fluid connection between clinical and basic researchers, and therefore, *translational medicine* is becoming increasingly prominent.

Progress in translational medicine crucially depends on close collaborations between three areas: technology development, research, and hospitals. This combination allows a research idea (or hypothesis) to be developed into a real concept (or product), thus producing substantial benefits to citizens. Such synergy between these areas is beginning to be found in health research institutes located in close vicinity to hospitals. These institutes usually house several core units, also known as *platforms* or *facilities*, which provide scientific support not only to their own researchers but also to hospitals and medtech companies.

Our cell culture platform participates in the three areas previously mentioned. On one hand, scientific, medical, or technological companies are constantly developing new products (such as cell culture dishes, factors, and antibodies) and advanced research tools. Prior to their commercialization, or in order to determine their efficacy, companies often need a network of scientific partners to test the products. The cell culture platform in our institute, for example, fostered by its physical proximity to technological companies, works as a service that tests those new products and tools.

On the other hand, research areas of the institutes require cell culture platform infrastructure in order to proceed with its research lines linked to cell cultures.

Hospitals are the third area related to the cell culture platform. Currently, the services offered by the cell culture platform are highly demanded by clinicians working in the hospital. For example, we assist in the expansion of clinical samples. Clinicians face difficulties to obtain large amounts of biological material (for example, blood samples of newborns, or amniotic liquid) in order to obtain DNA or RNA to perform a diagnosis. In undiagnosed newborns, pediatricians may decide to perform skin biopsies in order to establish cell cultures and this way, get enough DNA or RNA for the diagnosis. The cell culture platform offers all the basic equipment needed to process these biopsy samples, expand the cells *in vitro* and get enough genetic material for the genetic analysis. Moreover, these samples are deposited in the Biobank in order to be preserved for future diagnostic tests or for research purposes.

Additionally, increasing developments on cell transplantation-based therapies in hospitals is leading to hand-in-hand co-working between clinicians and scientists through cell culture platforms and is evoking an internal transformation. Currently, the therapeutic potential of several cell types is being tested in hospitals. Some examples include the injection of chondrocytes for bone regeneration, and the transplantation of human heart stem cells to treat myocardial infarctions.

Lately, innovative therapies based on the transplantation of extracellular vesicles (EVs) are emerging. In 2007, Valadi and colleagues (1) described a new mechanism of cells-to-cell communication consisting in the transfer of genetic material through EVs, which can be either intracellularly generated or extracellularly formed from the cell membrane. Actually, EVs have been detected in most body fluids, including blood and urine, and they have become a target to identify diagnostic biomarkers. During the last years, an increasing number of publications are demonstrating their importance, especially for exosomes (cell-derived vesicles of 30–100 nm of diameter) as prognostic markers or as therapeutic tools to treat cancer and vascular diseases (2, 3). EVs are optimal therapeutic vectors due to their ability to present MHC antigens, which prevents immune rejection when used in allogeneic transplantations (4). To date, three cell types – regulatory T-cells, mesenchymal stem cells (MSC), and dendritic cells – have been used as sources of EVs for transplantation. In order to standardize EV-based therapies, several parameters need to be controlled. These include, but are not limited to, EV extraction and processing protocols, EV identification protocols, purity assessment, route of administration, and doses.

From a clinical perspective, the performance of a clinical trial requires a quality control of the exosomes that are going to be injected in patients. This quality control should include at least a flow cytometry-based characterization, an ELISA-based quantification, and a nanoparticle tracking, besides purity assessment and exhaustive analysis to ensure the absence of mycoplasma, adventitious agents, and endotoxins (5).

The establishment of EV-based therapies will lead to standardized production and manipulation protocols following good manufacturing practices (GMPs), as well as quality controls to ensure the safety and efficacy of the treatments. Hence, how will these therapies impact cell culture platforms?

From my point of view, there could be two options for our platform, as cell extraction and culture, batch-production of EVs and packaging will have to be performed in GMP conditions: the first one is to adapt these requirements to a white room that will operate in line with GMP guidelines. That can be extremely costly. The second option will be to use the cell culture platform just as a service to perform EVs quality control whereas EVs obtaining from cells and packing is performed in a Contract Development and Manufacturing Organization (CDMO) specialized in the process development and GMP manufacture of cell therapy products from early stages up to clinical and commercial. In this case, the platform will only have to make a small investment to incorporate specific apparatus to determine EVs size and other EV-specific parameters.

In addition, even if the EVs production and packing is performed in an external CDMO, resuspension of the cryopreserved EVs can be performed in the platform in our biosecurity flow hoods. This way, the EVs delivery to the hospital, ready to inject, will be achieved a minimum time, as we are already operating with other cell therapies. This new challenge will have to be supported by specialized personnel in cell culture and EV related machinery as flow cytometry and EV related specific tools.

Bearing all this in mind if the cell culture platform would have to go toward EVs therapy, I would transform part of the platform in a “EVs sample quality service” (Figure 1). Otherwise, a big construction work with its high costs and complications would be difficult to face.

**Figure 1:**
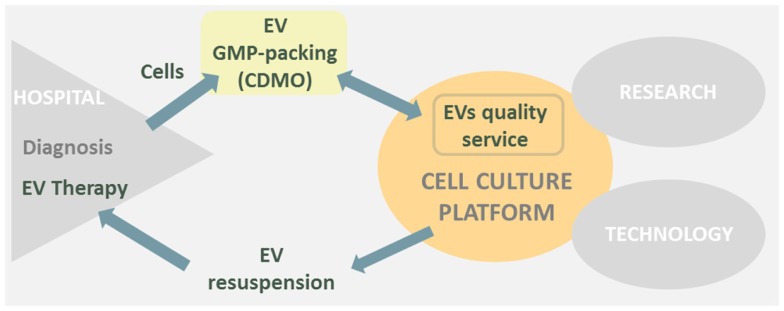
**Cell culture platform is placed in between research, hospital, and technological areas**. EVs therapies derived from hospitals could transform part of the cell culture platform in a EVs sample quality service and simultaneously EV resuspension point prior to human injection.

In conclusion, EVs therapies may have a great impact in the close future as new cell-based therapeutic approach; therefore, I will keep a sharp eye on EVs therapies future publications to envision the transformation of our cell culture platform toward a “EVs quality service.”

## Conflict of Interest Statement

The author declares that the research was conducted in the absence of any commercial or financial relationships that could be construed as a potential conflict of interest.
